# Disinfection by-products in drinking water and risk of colorectal cancer: a population-based cohort study

**DOI:** 10.1093/jnci/djad145

**Published:** 2023-08-08

**Authors:** Emilie Helte, Melle Säve-Söderbergh, Susanna C Larsson, Anna Martling, Agneta Åkesson

**Affiliations:** Unit of Cardiovascular and Nutritional Epidemiology, Institute of Environmental Medicine, Karolinska Institutet, Stockholm, Sweden; Unit of Cardiovascular and Nutritional Epidemiology, Institute of Environmental Medicine, Karolinska Institutet, Stockholm, Sweden; Science Division, Swedish Food Agency, Uppsala, Sweden; Unit of Cardiovascular and Nutritional Epidemiology, Institute of Environmental Medicine, Karolinska Institutet, Stockholm, Sweden; Unit of Medical Epidemiology, Department of Surgical Sciences, Uppsala University, Uppsala, Sweden; Department of Molecular Medicine and Surgery, Karolinska Institutet, Stockholm, Sweden; Department of Pelvic Cancer, GI Oncology and Colorectal Surgery Unit, Karolinska University Hospital, Stockholm, Sweden; Unit of Cardiovascular and Nutritional Epidemiology, Institute of Environmental Medicine, Karolinska Institutet, Stockholm, Sweden

## Abstract

**Background:**

Colorectal cancer is the third most common malignancy worldwide and is strongly linked to lifestyle and environmental risk factors. Although several drinking-water disinfection by-products are confirmed rodent carcinogens, the evidence in humans for carcinogenicity associated with these by-products, including colorectal cancer, is still inconclusive.

**Methods:**

We assessed the association of long-term exposure to trihalomethanes (THMs), the most prevalent disinfection by-products in chlorinated drinking water, with incidence of colorectal cancer in 58 672 men and women in 2 population-based cohorts. Exposure was assessed by combining long-term information of residential history with drinking water–monitoring data. Participants were categorized according to no exposure, low exposure (<15 µg/L), and high exposure (≥15 µg/L). Incident cases of colorectal cancer were ascertained by use of the Swedish National Cancer Register.

**Results:**

During an average follow-up of 16.8 years (988 144 person-years), 1913 cases of colorectal cancer were ascertained (1176 cases in men and 746 in women, respectively). High THM concentrations in drinking water (≥15 µg/L) were associated with increased risk of colorectal cancer in men (hazard ratio = 1.26, 95% confidence interval = 1.05-1.51) compared with no exposure. When subsites were assessed, the association was statistically significant for proximal colon cancer (hazard ratio = 1.59, 95% confidence interval = 1.11 to 2.27) but not for distal colon cancer or rectal cancer. In women, we observed overall no association of THMs with colorectal cancer.

**Conclusion:**

These results add further evidence that disinfection by-products in drinking water may be a possible risk factor for proximal colon cancer in men. This observation was made at THM concentrations lower than those in most previous studies.

Colorectal cancer is ranked as the third most common malignancy globally and is the second most common cause of cancer death. The incidence is about 4 times higher in transitioned countries than in transitioning countries, likely due to differences in lifestyle and exposure to environmental risk factors (1). Colorectal cancer is a heterogenous disease, with molecular cancer subtypes that are unevenly distributed along the colorectum (2). Proximal (right sided) and distal (left sided) colon cancers have distinct embryological origins, display different pathological and clinical features, and have been proposed to have different sensitivity toward environmental risk factors (3,4). In addition, although the incidence of overall colorectal cancer is higher in men, there is a female dominance in proximal colon cancer (5).

Disinfection by-products are reactive and potentially carcinogenic chemical substances that are formed when chlorine reacts with natural organic matter in drinking water. Trihalomethanes (THMs) are the class of by-products that are found at the highest concentrations in chlorinated drinking water, and several of these substances are genotoxic in vitro and rodent carcinogens (6). In carcinogenesis studies of rats, 2 of the most common THMs induced aberrant crypts and large-intestine carcinomas, which are anatomically and functionally analogous to colorectal cancer tumors in humans (7,8). In 2010, a meta-analysis summarized the epidemiological evidence for the association of disinfection by-products and colorectal cancer, and estimated that by-product exposure was associated with 27% and 30% increased odds of colon and rectal cancer, respectively (9). Nevertheless, the number of studies included was small, and each had important methodological limitations. In addition, although colorectal cancer is a highly heterogenous disease, to our knowledge no previous studies have investigated whether the association of colorectal cancer with THMs differs by subsites within the colon or rectum, and only a few studies have addressed potential differences associated with patient sex.

The aim of this study was to assess the association of exposure to disinfection by-products in drinking water, proxied by THMs concentrations, with incidence of colorectal cancer overall and according to subsites. We used data on middle-aged and older men and women (aged ≥65 years) enrolled in 2 large population-based cohorts in Sweden.

## Materials and methods

### Study population

The Swedish Mammography Cohort (SMC) and the Cohort of Swedish Men (COSM) are population-based longitudinal cohorts that are both part of the Swedish Infrastructure for Medical Population-based Life-course and Environmental Research (SIMPLER; www.simpler4health.se).

**Figure 1.**
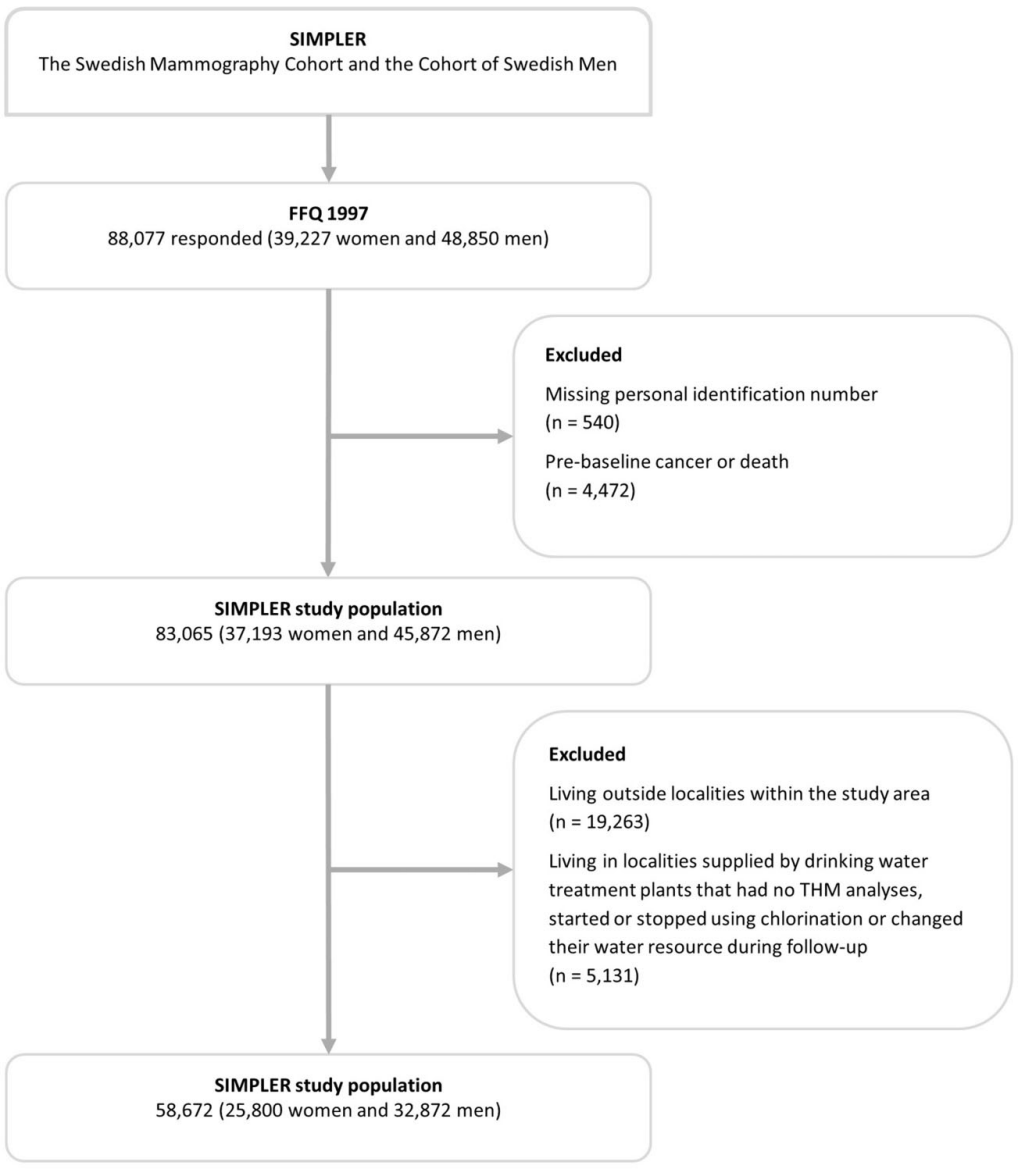
Flowchart of the study population.

The SMC was established in 1987-1990, when all women born in 1914-1948 in Uppsala County and 1917-1948 in Västmanland county were invited to participate in a mammography screening program (n = 90 303). Enclosed with the invitation was a questionnaire on diet and lifestyle (response rate = 74%). In 1997, a second questionnaire was sent to all women still living in the study area to update information on diet (96-item food frequency questionnaire) and health (response rate = 70%). After excluding women with incorrect or missing national registration number or history of or prevalent cancer disease (except nonmelanoma skin cancer), 37 193 women remained in the study (Figure 1).

Concurrent with the second female questionnaire being sent off, a cohort of male COSM participants was established. All men born in 1918-1952 and at the time living in Västmanland or Örebro counties received a questionnaire essentially identical to the 1997 SMC questionnaire, with the exception of some sex-specific questions (n = 100 303). In total, 49% of the invited men returned a completed questionnaire and after the respondents with incorrect or missing personal number or pre-existing cancer diagnosis, the male participant cohort consisted of 45 872 men (Figure 1). A comprehensive description of the cohorts is provided elsewhere (10).

In the present investigation, we included only participants who used drinking water from public drinking water systems. The rationale for not including participants whose drinking water was supplied by small private systems or private wells was that only public drinking water quality is closely monitored and reported to the national drinking water database Vattentäktsarkivet; Geological Survey of Sweden. Moreover, exclusion of participants whose drinking water does not come from public water systems reduces the risk of co-exposure or confounding by exposure to other waterborne constituents that may be less controlled in private drinking water. To identify those participants, we first gathered information on residential history from the National Register for Regional Divisions Based on Real Estate (Statistics Sweden) for all years available in the database (annual information, 1982-2019). We then excluded all participants living outside localities (defined as densely and coherent populated areas, with ≥1000 inhabitants) (n = 19 262, 23%) because these participants are likely not connected to the public drinking water system. Thereafter, we mapped all drinking water treatment plants distributing drinking water to the localities in the study area and collected information on their methods used for disinfection along with THM monitoring data from the Swedish Water analytical reports and Vattentäktsarkivet. If any alterations were made in the disinfection procedure or the water source changed during follow-up, the participants supplied by these treatment plants were excluded (n = 5129). This resulted in a final baseline study population of 58 672 participants (32 872 men and 25 800 women) (Figure 1).

The study was approved by the Regional Ethical Review Board in Stockholm, Sweden, and all subjects gave informed consent to participate in the study.

### Exposure assessment and covariates

#### Exposure assessment

We used the sum of the 4 most common THMs (chloroform, bromoform, dibromochloromethane, and bromodichloromethane) as a proxy of disinfection by-product exposure and gathered monitoring data (sampled in 2008-2017) collected in Vattentäktsarkivet. Conditioning on no changes having occurred in the drinking water treatment process, we extrapolated these concentrations back to the baseline in 1997, with the justification that little temporal variation is expected as long as disinfection schemes and water resources are unchanged. To account for seasonal variability in disinfection by-product formation (monitoring programs are often designed to sample for THMs more often in the spring and autumn when the concentrations peak) we calculated the average THM concentration per drinking water treatment plant for each season (December-February; March-May; June-August; September-November) and thereafter averaged these seasonal averages. In cases where more than 1 drinking water treatment plant supplied a locality, we used the average concentration of the supplying plants. The participants were then separated into exposure categories, determined based on previous knowledge regarding the distribution of THM concentrations in Swedish drinking water: zero exposure (no chlorination used in the treatment process), low THM exposure (<15 µg/L), and high THM exposure (≥15 µg/L). The categorization was based on participant residential addresses at baseline, and later updated annually so that participants were censored if they left the study area (ie, the exposure ceased or became unknown). In Sweden, tap water consumption is high, whereas bottled water consumption is very low (11,12), and therefore we assumed that THM exposure from drinking water was equivalent to that from tap water.

#### Covariates

Individual information on income and education was gathered from the Swedish Longitudinal Integrated Database for Health Insurance and Labour Market Studies register (Statistics Sweden), while self-reported information on smoking status, body mass index, and physical activity came from the baseline questionnaire. Information on dietary habits, including alcohol consumption, was computed from the 96-item food frequency questionnaire. Information on prevalent diabetes at baseline was based on self-reports and on data from the National Diabetes and Patient Registers (the National Board of Health and Welfare). Information on calcium in drinking water was based on monitoring data.

### Outcome assessment

Incident cases of colorectal cancer were ascertained from January 1, 1998, through a computerized linkage of the cohorts to the Swedish National Cancer Register. This register has more than 90% complete data for common cancers (13). The International Classification of Disease 10th revision (ICD-10) codes (14) C18-C20 were used to identify incident cases of colorectal cancer. Cases were further subdivided as follows: proximal colon cancer, that included tumors located in the cecum (C18.0), ascending colon (C18.2) and hepatic flexure (C18.3); distal colon cancer that included tumors the splenic flexure (C18.5), descending colon (C18.6), and sigmoid colon (C18.7); and rectal cancer (C20).

### Statistical analysis

We used Cox proportional hazards regression with attained age as the time scale to assess the association of exposure to THM and risk of colorectal cancer, generating hazard ratios (HRs) and 95% confidence intervals (CIs). Given the large disparities in disease incidence between men and women, both for colorectal cancer as a whole and per subsite, all analyses were stratified by sex (15). We calculated person time from January 1, 1998, until the date of first colorectal cancer diagnosis, date of death, date of moving from baseline living area (+2 years, because of a long induction time of cancer), or end of follow-up on December 31, 2020, whichever occurred first. In the analyses in which colorectal cancer subsites were assessed separately, participants were censored at any first cancer diagnosis in the colon or rectum, irrespective of the site under evaluation.

All multivariable adjusted models were adjusted for the following potential confounding factors, selected on the basis of being risk factors for colorectal cancer and potentially associated with THM exposure: level of education (<9, 9, 10-11, 12, and >12 years), household income (1000 SEK, quartiles) (16), smoking status (never, former <10, former ≥10, current <10, or current ≥10 cigarettes/day) (17), body mass index (<20, 20-24.9, 25-29.9, or ≥30 kg/m^2^) (18), diabetes (yes/no) (19), physical activity (walking >40 minutes/day or exercise >60 minutes/week, yes/no), alcohol intake (<5 g, 5-14.9, or ≥15 g/day), intake of red meat (servings/week, quartiles), intake of processed meat (servings/week, quartiles), intake of dairy products (servings/day, quartiles), intake of whole-grain foods (servings/week, quartiles), total energy intake (kcal/day, continuous), use of calcium supplements (yes/no), and drinking-water calcium (mg/L, continuous) (18). Missing information on covariates was handled by using a missing indicator category for categorical variables and by replacing continuous variables with the median. All covariates had less than 3% missing values except those proxying physical activity, which had on average 9% missing.

The proportional hazards assumption was tested using Schoenfeld residuals, and no indications of violations were observed. All tests were 2-sided, and the significance level was set at .05. The statistical software used was STATA/SE version 16.0 (StataCorp).

## Results

The mean (SD) drinking water THM concentration in the areas supplied by chlorinated drinking water was 10.7 (5.6) µg/L. No THMs were detected in drinking water in the areas supplied by drinking water treatment plants that used no chlorination. Age-standardized baseline characteristics of the study population are outlined in Table 1. There were no major differences in baseline characteristics across THM exposure groups apart from a higher proportion of women consuming ≥15 g of alcohol/day and a higher concentration of calcium in drinking water in the unexposed group compared with the exposed groups.

**Table 1.** Baseline age-standardized main characteristics of the 58 672 men and women in SIMPLER by cohort^a^

THM category	Men (COSM)			Women (SMC)		
	Nonchlorinated	Low THM (<15 µg/L)	High THM (≥15 µg/L)	Nonchlorinated	Low THM (<15 µg/L)	High THM (≥15 µg/L)
No. of participants	5785	18 505	9037	4534	11 376	9890
THM concentration, mean (SD), µg/L	—	6.8 (4.4)	15.5 (0.9)	—	6.2 (2.2)	18.5 (1.9)
Age, mean (SD), y	61 (10)	61 (10)	61 (10)	63 (9)	62 (9)	62 (10)
Education, y %						
≤9	40	36	35	48	43	33
10-11	21	20	21	32	30	26
≥12	39	44	45	21	27	41
Household income 1000 SEK/y, mean (SD)^b^	247 (131)	257 (151)	264 (378)	219 (210)	236 (151)	247 (165)
Smoking status, cigarettes/d, %						
Never	36	35	37	53	54	50
Former <10	13	14	14	11	12	13
Former ≥10	26	26	25	11	11	12
Current <10	11	11	10	11	10	12
Current ≥10	13	15	14	14	13	13
Drinking water calcium, mean (SD), mg/L	25 (7.8)	31 (6.3)	23 (2.3)	56.8 (20.1)	35.7 (16.8)	34.7 (6.8)
Prevalent diabetes, %	8	8	7	6	5	5
BMI, kg/cm^2^, mean (SD)	26 (3)	26 (3)	26 (3)	25 (4)	25 (4)	25 (4)
Physical activity, %						
Walk/bike ≥40 min/d	33	33	35	35	36	39
Exercise ≥1 h/wk	80	79	80	83	81	80
Alcohol, g/d, %						
≤0.5	39	35	33	73	58	63
0.5-15	41	41	41	24	28	32
>15	21	24	26	9	4	5
Intake of red meat, mean (SD), servings/wk	3.5 (2.6)	3.5 (2.9)	3.5 (2.7)	3.2 (2.3)	3.3 (2.6)	3.2 (2.4)
Intake of processed meat, mean (SD), servings/wk	5.7 (4.6)	5.5 (4.6)	5.8 (4.8)	4.6 (4.1)	4.7 (4.3)	4.2 (3.9)
Intake of whole-grain, mean (SD), servings/wk	4.7 (2.6)	4.5 (2.5)	4.4 (2.6)	4.1 (2.0)	4.1 (2.2)	4.1 (2.1)
Intake of dairy products, mean (SD), servings/d	5.1 (3.0)	5.1 (3.0)	5.2 (3.0)	4.6 (2.7)	4.7 (2.8)	4.7 (2.8)
Total energy intake, mean (SD), kcal/d	2600 (882)	2583 (889)	2588 (870)	1695 (568)	1720 (589)	1725 (572)
Use of calcium supplements, %	2	2	2	6	6	8

a The sum of chloroform, bromoform, dibromochloromethane, and bromodichloromethane. BMI = body mass index; COSM = cohort of Swedish men; SD = standard deviation; SMC = Swedish Mammography Cohort; THM = trihalomethanes.

b 1000 SEK = 100 EUR or 121 USD (exchange rate February 2021).

Over an average follow-up of 16.8 years (988 144 person-years), we ascertained 1913 cases of any first colorectal cancer (1176 men and 746 women). When cases were subdivided into proximal colon cancer, distal colon cancer and rectal cancer the number of first cases were 633 (315 men, 318 women), 509 (327 men, 182 women), and 630 (435 men, 195 women), respectively.

Drinking water THM was associated with an increased risk of colorectal cancer among men, with a multivariable adjusted HR of 1.26 (95% CI = 1.05 to 1.51, Table 2) for the group with highest exposure compared with the unexposed group. No association was observed among women (HR = 0.97, 95% CI = 0.77 to 1.23, Table 2). In subsite analyses, we observed increased risk of proximal colon cancer among the men exposed to ≥15 µg/L drinking-water THM compared with the unexposed men (HR = 1.59, 95% CI = 1.11 to 2.27), although no clear association was observed for distal colon and rectal cancer (Table 3). Among women, THM in drinking water was not associated with risk of cancer at any subsite of the colorectum (Table 4).

**Table 2.** Hazard ratios of colorectal cancer by THM exposure categories in the 58 672 men and women in SIMPLER stratified by sex^a^

THM	Non-chlorinated	Low THM (<15 µg/L)	High THM (≥15 µg/L)
Men, n = 32 872			
No. of participants	5785	18 050	9037
Cases	176	650	341
Person-years	94 558	294 357	147 707
Age-adjusted HR (95% CI)^b^	1.00	1.20 (1.02 to 1.42)	1.26 (1.05 to 1.51)
Multivariable adjusted HR (95% CI)^e^	1.00	1.23 (1.03 to 1.47)	1.26 (1.05 to 1.51)
Women, n = 25 800			
No. of participants	4534	11 376	9890
Cases	136	322	288
Person-years	77 702	199 926	173 960
Age-adjusted HR (95% CI)^b^	1.00	0.93 (0.76 to 1.14)	0.97 (0.79 to 1.19)
Multivariable adjusted HR (95% CI)^c^	1.00	0.93 (0.74 to 1.17)	0.97 (0.77 to 1.23)

a The sum of chloroform, bromoform, dibromochloromethane, and bromodichloromethane. CI = confidence interval; HR = hazard ratio; THM = trihalomethanes.

b Adjusted for age (underlying timescale) and cohort (stratum variable).

c Further adjusted for level of education (<9, 9, 10-11, 12, and >12 years), household income (1000 SEK, quartiles), smoking status (never, former <10, former ≥10, current <10, or current ≥10 cigarettes/day), body mass index (<20, 20-24.9, 25-29.9, or ≥30 kg/m^2^), diabetes (yes/no), physical activity (walking >40 minutes/day or exercise >60 minutes/week, yes/no), alcohol intake (<5, 5-14.9, or ≥15 g/day), intake of red meat (servings/week, quartiles), intake of processed meat (servings/week, quartiles), intake of dairy products (servings/day, quartiles), intake of whole-grain foods (servings/week, quartiles), total energy intake (kcal/day, continuous), use of calcium supplements (yes/no), and drinking-water calcium (mg/L, continuous).

**Table 3.** Hazard ratios of proximal colon cancer, distal colon cancer, and rectal cancer by THM exposure categories in the 32 872 men in COSM^a^

THM	Non-chlorinated	Low THM (<15 µg/L)	High THM (≥ 15 µg/L)
No. of participants	5785	18 050	9037
Person-years	94 558	294 357	147 707
Proximal colon cancer			
No. of cases	43	167	105
Age-adjusted HR (95% CI)^b^	1.00	1.26 (0.90 to 1.76)	1.57 (1.10 to 2.24)
Multivariable adjusted HR (95% CI)^c^	1.00	1.27 (0.88 to 1.83)	1.59 (1.11 to 2.27)
Distal colon cancer			
Cases	49	194	84
Age-adjusted HR (95% CI)^b^	1.00	1.29 (0.94 to 1.76)	1.11 (0.78 to 1.58)
Multivariable adjusted HR (95% CI)^c^	1.00	1.16 (0.83 to 1.62)	1.14 (0.80 to 1.63)
Rectal cancer			
Cases	71	238	126
Age-adjusted HR (95% CI)^b^	1.00	1.09 (0.84 to 1.42)	1.15 (0.86 to 1.54)
Multivariable adjusted HR (95% CI)^c^	1.00	1.22 (0.91 to 1.64)	1.16 (0.86 to 1.55)

a The sum of chloroform, bromoform, dibromochloromethane, and bromodichloromethane. CI = confidence interval; COSM = cohort of Swedish men; HR = hazard ratio; THM = trihalomethane.

b Adjusted for age (underlying timescale).

c Further adjusted for level of education (<9, 9, 10-11, 12, and >12 years), household income (quartiles), smoking status (never, former <10, former ≥10, current <10, current ≥10 cigarettes/d), BMI (<20, 20-24.9, 25-29.9 , or ≥30 kg/m^2^), diabetes (yes/no), physical activity (walking >40 min/d or exercise >60 min/wk, yes/no), alcohol intake (<5 , 5-14.9, or ≥15 g/d), intake of red meat (quartiles), intake of processed meat (quartiles), intake of dairy products (quartiles), intake of whole grains (quartiles), total energy intake (continuous, kcal), use of calcium supplements (yes/no), drinking-water calcium (continuous, mg/L).

**Table 4.** Hazard ratios of proximal colon cancer, distal colon cancer, and rectal cancer by THM exposure categories in the 25 800 women in the SMC

THM^a^	Non-chlorinated	Low THM (<15 µg/L)	High THM (≥ 15 µg/L)
No. of participants	4534	11 376	9890
Person-years	77 702	199 926	173 960
Proximal colon cancer			
Cases	57	138	123
Age-adjusted HR^b^ (95% CI)	1.00	0.96 (0.71 to 1.31)	0.99 (0.72 to 1.35)
Multivariable adjusted HR^c^ (95% CI)	1.00	0.92 (0.65 to 1.31)	0.95 (0.66 to 1.36)
Distal colon cancer			
Cases	25	81	76
Age-adjusted HR^b^ (95% CI)	1.00	1.27 (0.81 to 2.00)	1.39 (0.88 to 2.18)
Multivariable adjusted HR^c^ (95% CI)	1.00	1.18 (0.71 to 1.96)	1.30 (0.78 to 2.18)
Rectal cancer			
Cases	45	79	71
Age-adjusted HR^b^ (95% CI)	1.00	0.69 (0.48 to 0.99)	0.72 (0.49 to 1.04)
Multivariable adjusted HR^c^ (95% CI)	1.00	0.81 (0.53 to 1.23)	0.85 (0.54 to 1.32)

a The sum of chloroform, bromoform, dibromochloromethane, and bromodichloromethane. CI = confidence interval; HR = hazard ratio; SMC = Swedish Mammography Cohort; THM = trihalomethane.

b Adjusted for age (underlying timescale).

c Further adjusted for level of education (<9, 9, 10-11, 12, and >12 years), household income (quartiles), smoking status (never, former <10, former ≥10, current <10, current ≥10 cigarettes/d), BMI (<20, 20-24.9, 25-29.9, or ≥30 kg/m^2^), diabetes (yes/no), physical activity (walking >40 min/d or exercise >60 min/wk, yes/no), alcohol intake (<5, 5-14.9, or ≥15 g/d), intake of red meat (quartiles), intake of processed meat (quartiles), intake of dairy products (quartiles), intake of whole grains (quartiles), total energy intake (continuous, kcal), use of calcium supplements (yes/no), drinking-water calcium (continuous, mg/L).

## Discussion

In this population-based cohort of middle-aged to older men and women, we observed an association of drinking water with THM concentrations more than 15 µg/L, with 59% (95% CI = 11% to 127%) elevated risk of proximal colon cancer in men compared with men who drank non-chlorinated water. No clear association was observed for distal colon cancer and rectal cancer. In women, we observed overall no association of drinking water THM with colorectal cancer. These data add support to the notion of disinfection by-products in drinking water as a possible risk factor for proximal colon cancer in men.

Our findings of an association of THMs in drinking water with an increased risk of proximal cancer are in line with previous research findings. In 2010, epidemiological evidence summarized in a meta-analysis indicated that disinfection by-product exposure was associated with 27% increased odds of colon cancer (9). Since then, a few more studies on this topic have been published. In a multicenter case-control study in Spain, residential drinking water concentrations of brominated THMs were associated with increased risk of colorectal cancer in men, although not statistically significant (odds ratio [OR] = 1.43, 95% CI = 0.83 to 2.46) (20). These findings accord well with the results of an ecological analysis in Australia, in which increased incidence rates of colorectal cancer, particularly colon cancer, were observed in men but not women in relation to bromoform concentrations in residential drinking water (21). Moreover, based on the findings of a case-control study in Ethiopia, living in a household supplied with chlorinated surface water was estimated to be associated with increased odds of colorectal cancer (OR = 2.6, 95% CI = 1.7 to 4.0) (22). In contrast to our findings of no association of THMs with colorectal cancer in women, increasing drinking-water THM concentrations were associated with risk of rectal cancer (HR = 1.71, 95% CI = 1.00 to 2.92) in the Iowa Women’s Health cohort, but no clear association was observed with risk of colon cancer (HR = 1.13, 95% CI = 0.89 to 1.44) (23). Several reasons may underlie the contrasting findings on rectal cancer, including slightly higher concentrations of THMs in drinking water in the American study or the use of different chlorination schemes resulting in disinfection by-product mixtures with somewhat dissimilar compositions.

Given that the individual THMs exhibit slightly different toxicological properties, some studies have examined these separately in relation to colorectal cancer risk. In a case-control study of men in Western New York State, USA, only the brominated THMs were associated with increased risk of rectal cancer (24). Similarly, in a Spanish case-control study in which brominated THMs were associated with increased risk of colorectal cancer in men, no association was observed with total THMs, and an inverse association was observed with chloroform (20). Similar differences have been found in experimental studies and suggest that not all individual THMs are equally toxic (6). The proportion of individual THMs in different disinfection by-product mixtures depends on a number of factors, including the levels of bromine in the raw water, which highlights the complexity and challenges associated with studying the health effects of these mixtures.

In addition to the epidemiological evidence, data from mechanistic and animal studies support our observation of an association of THMs with increased risk of proximal colon cancer. The 3 brominated THMs are all genotoxic in standard test systems after glutathione S-transferase theta 1 (GSTT1-1) biotransformation. Although chloroform is not genotoxic, it induces tumors in rodent carcinogenicity bioassays (25), presumably through pathways involving cytotoxicity and regenerative cell proliferation (6). Interestingly, bromoform and bromodichloromethane, which are also the disinfection by-products that have been the most consistently associated with increased colorectal cancer risk in humans, have been shown to produce aberrant crypts and tumors of the large intestine in rats (7,8). It should be noted that these tumors are anatomically and functionally analogous to those in human colorectal cancer (6), and historically very rare (<0.2%) in the rat strain tested (7). Along with the other THMs, chlorodibromomethane is also a rodent carcinogen, although mainly associated with hepatocellular carcinoma (26).

In our study, we found that the association of drinking water THMs and colorectal cancer was dissimilar between men and women, and that it varied by tumor location. These differences by anatomical location may be attributable to the uneven distribution of molecular subtypes of colorectal cancer along the colon, which has distinct etiology and may exhibit different sensitivity toward environmental risk factors (3,4). In addition, men and women have different colorectal cancer risk levels, so that although men have a higher overall risk for colorectal cancer, proximal colon cancer is more prevalent in women (27). Sex differences in cancer risk following THM exposure have also been observed in animals under experimental conditions (8).

Disinfection by-product exposure has also been evaluated in relation to several other forms of cancer, including (but not limited to) bladder cancer (28), kidney cancer (29), and pancreatic cancer (30). Most evidence for an association with increased cancer risk in humans exists for bladder cancer, and the most recent meta-analysis estimated 47% increased odds of bladder cancer for men exposed to THM concentrations above 50 µg/L compared with men exposed to 5 µg/L or less (28).

This study has several strengths. First, we employed a population-based prospective cohort design, including both male and female participants. Second, the computerized linkage to several high-quality registers enabled an almost complete ascertainment of cases and provided detailed information on residential history as well as on a number of potential confounding factors related to socioeconomics. Last, from the questionnaires we obtained detailed information on several other important risk factors for colorectal cancer, including diet.

Some study limitations also need to be acknowledged. First, THM formation is complex and varies with season, temperature, pH, and retention time in the distribution system, making some degree of exposure misclassification inevitable. Moreover, the exposure was assigned on the basis of the concentration of THMs in drinking water at the participants’ homes, assuming that their drinking water consumption at locations supplied by different drinking water treatment plants was minimal. Moreover, we did not have information on individual tap water consumption or bottled water consumption, and therefore we were unable to estimate associations of the total amount THM ingested or correct for differences in bottled water consumption across THM categories. Last, we used THMs as a proxy for general disinfection by-product exposure, and in some cases (ie, when chlorine is not the main disinfectant), THMs are poor indicators for the exposure. In addition, we had limited data on the individual THMs and were not able to assess the associations of these with colorectal cancer risk separately. Given that disinfection by-products are a complex mixture of more than 600 identified substances, any associations of THMs with risk of colorectal cancer and subsites observed in our study could, partly or entirely, be explained by other coexisting by-product substances, for example, haloacetic acids, or be due to interaction with other substances in the disinfection by-product mixture (31,32).

In conclusion, in this population-based cohort of middle-aged to older men and women, we observed an association of THM in drinking water with increased risk of proximal colon cancer that was restricted to men. This observation was made at THM concentrations lower than in most previous studies. These data add support to previous indications of disinfection by-products in drinking water as a possible risk factor for proximal colon cancer in men.

## Data Availability

The data underlying this article cannot be shared publicly because it contains sensitive personal information that must be stored and handled according to the EU Regulation 2016/679 General Data Protection Regulation (GDPR). Researchers who meet the criteria for access to SIMPLER data (ie, ethical approval is required) can apply for cohort data from the SIMPLER web page (https://www.simpler4health.se). Data from Statistics Sweden linked to cohort data can also be applied for from Statistics Sweden through SIMPLER. Drinking water monitoring data can be applied for from the Geological Survey of Sweden.
